# The use of patient-generated health data in the management of low anterior resection syndrome: a qualitative study

**DOI:** 10.3389/fsurg.2024.1506688

**Published:** 2024-12-19

**Authors:** Olivia Monton, Allister Smith, Sarah Sabboobeh, Marie Demian, Julie Cornish, Steven D. Wexner, Peter Christensen, Amandeep Ghuman, Liliana G. Bordeianou, Celia Keane, Syed Husain, Alessandra Gasior, Natalie Leon, Julie Savard, Lieba R. Savitt, Margit Majgaard, Gitte Kjær Sørensen, Melanie Mills, Fateme Rajabiyazdi, Marylise Boutros

**Affiliations:** ^1^ Department of Surgery, McMaster University, Hamilton, ON, Canada; ^2^ Department of Epidemiology, Johns Hopkins Bloomberg School of Public Health, Baltimore, MD, United States; ^3^ Department of Emergency Medicine, Western University, London, ON, Canada; ^4^ Department of Surgery, Division of Colon and Rectal Surgery, Jewish General Hospital, Montreal, QC, Canada; ^5^ Cardiff and Vale University Health Board, Cardiff University, Cardiff, United Kingdom; ^6^ Department of Colorectal Surgery, Cleveland Clinic Florida, Weston, FL, United States; ^7^ Aarhus University Hospital, Aarhus, Denmark; ^8^ Danish Cancer Society National Research Centre for Survivorship and Late Adverse Effects Following Pelvic Organ Cancer, Aarhus, Denmark; ^9^ Department of Surgery, Division of Colon and Rectal Surgery, St. Paul’s Hospital, Vancouver, BC, Canada; ^10^ Department of Colorectal Surgery, Massachusetts General Hospital, Boston, MA, United States; ^11^ Center for Pelvic Floor Disorders, Massachusetts General Hospital, Boston, MA, United States; ^12^ Harvard Medical School, Boston, MA, United States; ^13^ Department of Surgery, Faculty of Medical and Health Sciences, University of Auckland, Auckland, New Zealand; ^14^ Department of Surgery, Whangarei Hospital, Te Whatu Ora—Health New Zealand—Te Tai Tokerau, Whangārei, New Zealand; ^15^ The Ohio State University, Columbus, OH, United States; ^16^ The Ohio State Wexner Medical Center, Columbus, OH, United States; ^17^ Nationwide Children’s Hospital, Columbus, OH, United States; ^18^ Department of Systems and Computer Engineering, Carleton University, Ottawa, ON, Canada; ^19^ Department of Surgery, McGill University, Montreal, QC, Canada

**Keywords:** low anterior resection, low anterior resection syndrome (LARS), patient-generated health data (PGHD), colorectal surgery, self-management

## Abstract

**Background:**

The cornerstone of low anterior resection syndrome (LARS) treatment is self-management, which requires patient engagement. Colorectal surgeons and nurses may use patient-generated health data (PGHD) to help guide patients in their use of self-management strategies for LARS. However, the perspectives of LARS experts on the use of PGHD remain largely unexplored. The objective of this study was to explore the perspectives and experiences of LARS experts regarding the use of PGHD in the management of LARS.

**Methods:**

We utilized purposive snowball sampling to identify international LARS experts, including surgeons, nurses, and LARS researchers with knowledge and expertise in LARS. We conducted individual semi-structured interviews with these experts between August 2022 and February 2024. We performed thematic analysis using the framework method to identify domains and associated themes.

**Results:**

Our sample included 16 LARS experts from five countries. Thematic analysis identified four domains and associated themes. The domains included: data collection practices, data review practices, perceived usefulness, and future directions. Within the data collection practices domain, we found that most experts asked LARS patients to collect some form of PGHD, including bowel diaries, patient-reported outcome measures, or both. Within the data review practices domain, we found that both surgeons and nurses reviewed PGHD. Most participants described finding it difficult to interpret the data and identified time constraints, legibility, and completeness as the most common barriers to reviewing data in clinic. In terms of perceived usefulness, data collection was felt to help clinicians understand symptoms and their impact and assist patients with self-management. The future directions domain revealed that most experts felt that a clinical tool in the form of an online app or website to support data collection and enhance data visualization would be useful. Finally, some participants saw promise in leveraging PGHD to inform the creation of automated treatment algorithms for LARS management.

**Conclusions:**

This study highlights many gaps in the processes of patient-generated LARS data collection and review. A clinical tool including various data collection templates and data visualization prototypes could help to address these gaps. Future research will focus on incorporating the patient perspective.

## Introduction

Low anterior resection syndrome (LARS) is a significant sequelae of rectal cancer treatment and has important implications for patients. LARS is defined as a constellation of negative bowel symptoms, including fecal incontinence, urgency, frequency, and clustering of bowel movements (1, 2). Up to 70% of patients who undergo restorative proctectomy for the treatment of rectal cancer develop this long-term bowel dysfunction, which has been shown to have a negative impact on patient wellbeing and quality of life (1–6). Current management of LARS is empirical and symptom-based, requiring a large degree of patient engagement and self-management. In the context of chronic disease, self-management tasks can be understood as a combination of medical management, maintaining, changing, and adopting new behaviors, and addressing the emotional consequences of living with a chronic condition (7, 8).

Patient-generated health data (PGHD) refers to “health-related data created, recorded, or gathered by or from patients to help address a health concern.” (9) PGHD is increasingly utilized in the treatment of chronic diseases to provide self-management support (10). Various tools are available to facilitate the collection and review of PGHD across a range of chronic conditions, including monitoring blood sugar levels (11, 12), sleep patterns (13), weight management (14–16), and cancer care (17). These tools may include a combination of diary entries with self-reported health information, patient-reported outcome measures (PROMs) used in clinical practice, and biometric sensor data, depending on the setting and context. These tools may also include visual summaries in the form of graphical representations, with the goal of helping patients and clinicians visualize health data and identify trends over time. In many settings, the use of PGHD has been found to enhance health awareness, patient motivation and engagement, and patient-clinician communication (18, 19).

As with other chronic diseases, the use of PGHD following restorative proctectomy has the potential to improve LARS management, however, there remains considerable variablility in its use in both patient care and research. In their recent work, Garfinkle et al. incorporated bowel symptom, diet, and loperamide diaries into a LARS patient-centred programme (LPCP), which is currently being evaluated through a randomized controlled trial (20). The authors highlighted that the goal of these diaries was to help patients recognize patterns related to their symptoms to optimize self-management. Whereas, Harji et al. incorporated PROMs, including the LARS score and Wexner Faecal Incontinence score, to gather granular data on symptom burden within a bowel rehabilitation programme (BOREAL), which involved a stepwise approach to escalating treatments for LARS patients (21). The authors demonstrated the acceptability, feasibility, and effectiveness of the program, highlighting the successful integration of clinical, oncological, and functional assessments.

While these examples provide insight into differing approaches to the use of PGHD in LARS research, with implications for patient care, literature on the utilization of PGHD in LARS management remains limited and has not been directly investigated or reported. Similarly, in clinical practice, the use of PGHD in LARS management is inconsistent and sporadic across colorectal surgery practices, with no consensus on its optimal application. The goal of this study was to explore the perspectives and experiences of LARS experts—including colorectal surgeons, nurses, and researchers—on the use of PGHD in the management of LARS. Specifically, the study aimed to answer the following research questions:

1. How are LARS experts currently incorporating PGHD into the management of LARS?
2. What are the perceived barriers and facilitators to the use of PGHD in this context?
3. What are LARS experts' perspectives on the potential impact of PGHD on patient outcomes and self-management?

## Materials and methods

This was a qualitative study conducted using individual semi-structured interviews with professional key informants, who had expertise in the management of LARS, from Canada, the United States, the United Kingdom, Denmark, and New Zealand. This study is reported in accordance with the consolidated criteria for reporting qualitative studies (COREQ) checklist (Table 1) (22).

**Table 1:** Consolidated criteria for reporting qualitative studies (COREQ) checklist.

No	Item	Guide questions/description	Item reported?
Domain 1: Research team and reflexivity			
Personal characteristics (methods section; data collection)			
1	Interviewer/facilitator	Which author/s conducted the interview or focus group?	Yes
2	Credentials	What were the researcher’s credentials? E.g. PhD, MD	Yes
3	Occupation	What was their occupation at the time of the study?	Yes
4	Gender	Was the researcher male or female?	Yes
5	Experience and training	What experience or training did the researcher have?	Yes
Relationship with participants (methods; data collection)			
6	Relationship established	Was a relationship established prior to study commencement?	Yes
7	Participant knowledge of the interviewer	What did the participants know about the researcher? E.g. personal goals, reasons for doing the research	Yes
8	Interviewer characteristics	What characteristics were reported about the interviewer/facilitator? E.g. Bias, assumptions, reasons and interests in the research topic	No
Domain 2: study design			
Theoretical framework			
9	Methodological orientation and theory	What methodological orientation was stated to underpin the study? E.g. grounded theory, discourse analysis, ethnography, phenomenology, content analysis	Yes
Participant selection			
10	Sampling	How were participants selected? E.g. purposive, convenience, consecutive, snowball	Yes
11	Method of approach	How were participants approached? E.g. face-to-face, telephone, mail, email	Yes
12	Sample size	How many participants were in the study?	Yes
13	Non-participation	How many people refused to participate or dropped out? Reasons?	No
Setting (data collection in the methods section			
14	Setting of data collection	Where was the data collected? E.g. home, clinic, workplace.	Yes
15	Presence of non-participants	Was anyone else present besides the participants and researchers?	Yes
16	Description of sample	What are the important characteristics of the sample? E.g. demographic data, date	Yes
Data collection			
17	Interview guide	Were questions, prompts, guides provided by the authors? Was it pilot tested?	Yes
18	Repeat interviews	Were repeat interviews carried out? If yes, how many?	No
19	Audio/visual recording	Did the research use audio or visual recording to collect the data?	Yes
20	Field notes	Were field notes made during and/or after the interview or focus group?	No
21	Duration	What was the duration of the interviews or focus group?	Yes
22	Data saturation	Was data saturation discussed?	No
23	Transcripts returned	Were transcripts returned to participants for comment and/or correction?	No
Domain 3: analysis and findings			
Data analysis			
24	Number of data coders	How many data coders coded the data?	Yes
25	Description of the coding tree	Did authors provide a description of the coding tree?	Yes
26	Derivation of themes	Were themes identified in advance or derived from the data?	Yes
27	Software	What software, if applicable, was used to manage the data?	Yes
28	Participant checking	Did participants provide feedback on the findings?	No
Reporting (results)			
29	Quotations presented	Were participant quotations presented to illustrate the themes/findings? Was each quotation identified? e.g. participant number	Yes
30	Data and findings consistent	Was there consistency between the data presented and the findings?	Yes
31	Clarity of major themes	Were major themes clearly presented in the findings?	Yes
32	Clarity of minor themes	Is there a description of diverse cases or discussion of minor themes?	Yes

### Participants and recruitment

We used purposive snowball sampling to identify international LARS experts through a group of international LARS collaborators. Participants were eligible to participate if they: (1) had an academic appointment as a surgeon, nurse, or interacted with patients as a LARS researcher; (2) had expertise in LARS (clinical or research), and (3) were English speaking. The senior author (MB) contacted eligible participants via email through an established network of international LARS collaborators to introduce the study and solicit participation. The lead author (OM) then emailed those interested to describe the study in detail and schedule interviews at mutually agreeable times.

### Interview guide

The central research team comprised two resident physicians (OM, AS), one colorectal surgeon (MB), two research associates (SS, MD), and one qualitative methods expert (FR). The semi-structured interview guide was created by members of the research team with expertise in LARS (OM, MB) and qualitative research (FR). The interview guide spanned several key content areas, including experiences with the use of PGHD in the management of LARS (e.g., data collection and review practices), the potential benefits and challenges associated with the use of patient-generated LARS data, and perspectives on its optimal use for LARS patients. We created three versions of the interview guide for the different types of professional key informants, including colorectal surgeons, LARS nurses, and LARS researchers. This approach ensured that the questions were tailored to the expertise of each type of professional key informant. The semi-structured interview guides are available in Supplementary Material, Table A1.

### Interview procedures

The interviews were conducted between August 2022 and February 2024 using a secure version of the Zoom platform (Zoom Video Communications, Inc., San Jose, CA). Each interview lasted approximately 30–45 min and was performed by one author (OM), a female general surgery resident who completed formal training in qualitative research at the time of the interviews. The interviews were audio-recorded.

### Analysis

The audio recordings were transcribed verbatim, checked for accuracy, and imported into the MAXQDA Version 2020 software (VERBI Software, Berlin, Germany). All transcripts were anonymized and labeled, including a key informant number (i.e., KI01-KI16) and type of informant (i.e., colorectal surgeon, nurse, researcher). We performed thematic analysis using the framework methodology outlined by Gale et al. (23). After a process of familiarization, two authors (OM, AS) created the codebook using an abductive approach, balancing the development of deductive codes generated from the interview guide with inductive codes that were identified from the data (24). The two authors developed a preliminary codebook and independently coded five transcripts. Subsequently, they adapted and refined the codebook based on patterns and themes that emerged from the data. Once consensus was reached, one author (OM) independently coded all of the transcripts. Disagreements throughout the coding process were resolved through discussion with two authors (FR, MB). Following coding, the authors identified four key content areas that emerged throughout the analytic process: data collection, data review, data utility, and future directions. These content areas served as the thematic framework for an analysis table. The coded segments were summarized and added to the analysis table. Four authors (OM, AS, FR, MB) then employed the constant comparison method to generate and refine domains and associated themes.

## Results

Our sample comprised 16 international LARS experts, including 8 surgeons, 6 nurses, and 2 research associates. All surgeons were specialty-trained in colorectal surgery, with several years of experience working with LARS patients, both in clinical and research capacities. Each nurse worked directly alongside an interviewed colorectal surgeon, was clinically trained in managing LARS patients, and possessed substantial clinical expertise in this area. The two research associates interviewed held university-affiliated research positions, where they worked closely with LARS patients in research-focused roles. Participants were from Montreal (*n* = 4, 25%) and Vancouver (*n* = 1, 6.25%), Canada; Massachaussettes (*n* = 2, 12.5%), Ohio (*n* = 2, 12.5%), and Florida (*n* = 1, 6.25%), United States; Cardiff, United Kingdom (*n* = 2, 12.5%); Aarhus, Denmark (*n* = 3, 18.75%); and Auckland, New Zealand (*n* = 1, 6.25%). There were 13 (81.25%) females and 3 (18.75%) males. Most participants (*n* = 15, 93.75%) reported working in academic practice settings, and 1 participant reported working in a community hospital (*n* = 1, 6.25%). Participants reported being in practice for a median of 17.5 years (IQR: 9.5–25.5). Informants attested to working with LARS patients for a median of 10 years (IQR: 7.5–14.5).

**Figure 1:**
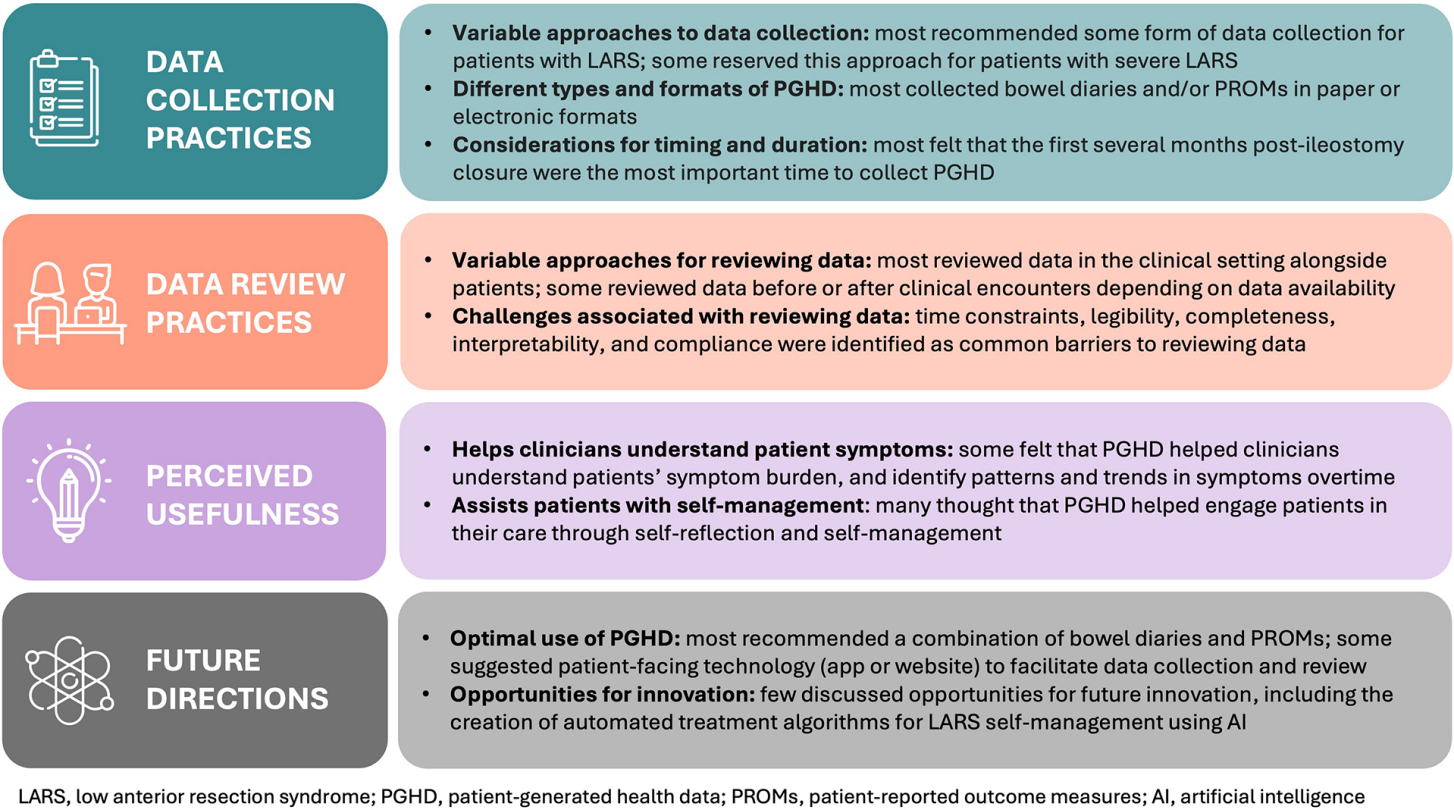
Summary of findings organized by domain.

Our thematic analysis revealed four domains: data collection practices, data review practices, perceived usefulness, and future directions, with several associated themes. A summary of the findings is outlined in Figure 1.

### Data collection practices

#### Variable approaches to data collection

Participants described variable approaches to data collection. Most of the informants we interviewed recommended that patients experiencing LARS symptoms collect some form of PGHD. However, not all informants reported collecting PGHD routinely on all patients. Some reserved this approach for patients with severe LARS or for those who were poor historians and were unable to articulate their symptoms. Participants perceived patients with severe symptoms as being more motivated to collect their data compared to patients with less severe or no symptoms. KI11, a LARS Nurse, stated: “I think it depends on how motivated they are, right? Like if they're miserable, they're more willing to put the time in because they want to see improvements.” Despite the use of PGHD within their own individual practices, or in some cases institutional or regional settings, many informants perceived a lack of standardization in the use of PGHD across colorectal surgery practices broadly.

Data collection was perceived as being clinician-initiated in most cases with clinicians recommending that patients collect some form of PGHD (see examples below), however, some informants noted that few patients collect their own data independently, without prompting by the healthcare team. One informant highlighted the potential role of anxiety as a driver for patient-initiated data collection:

In the last four years, ninety percent of it is initiated on my part. Patients rarely come in with that information, unless they are an anxious individual, and they will note, “this is how I know how to manage my anxiety, is to keep track of stuff.” But ninety percent of the time it will be initiated by myself.—KI14, LARS Nurse

Another participant identified that patients who were “data driven” tended to be more likely to collect data independently: “Some patients are very objective and data driven. I have a few of those, and like I said, those are the ones that usually bring in the notebooks and have all the information” (KI05, Colorectal Surgeon). This sentiment was shared by another surgeon, who expressed similar patterns within their practice, describing a patient with a background in engineering, who approached data collection in an analytical manner: “I had an engineer once that made up a scale from 1 to 10 and had a little graph with an X and a Y. So yeah, some people go above and beyond” (KI03, Colorectal Surgeon).

#### Different types and formats of PGHD

Participants attested to asking patients to collect different types of PGHD, including bowel diaries, PROMs, or a combination of both. Those who asked patients to keep bowel diaries typically had patients collect granular information on bowel movements, diet, and medication use, including a combination of symptom characteristics (frequency, timing, urgency, consistency, and volume of bowel movements) and severity; potential triggers; changes in symptom burden associated with medications or interventional strategies; and impact on quality of life. KI03, a Colorectal Surgeon, highlighted the importance of capturing a combination of symptom characteristics and impact on quality of life:

If you are just recording the frequency of accidents, you are barely scratching the surface of what they are actually experiencing. Because there is so much more that happens [with LARS]. And the biggest complaint that patients have is not so much the accidents but the fragmentation of the bowel movements, the clustering of the bowel movements, the pain with bowel movements, the hoops they have to jump through to address the urgency of their bowel movements and the social inconveniences that are associated with it.—KI03, Colorectal Surgeon

This informant underscored the importance of understanding how patients experienced their symptoms, with an emphasis on symptom impact on quality of life and daily functioning. Many participants similarly expressed the importance of capturing the degree to which LARS symptoms were impacting patients' physical, mental, and social wellbeing and functioning. This would help them identify patients' goals and ensure that management strategies were appropriately aligned with their goals.

Some informants also collected PROMs, and endorsed using them for clinical practice. Most commonly, these included measures of bowel function and quality of life, including: (1) the LARS score, a symptom-based scoring system for bowel dysfunction after surgery (6); (2) the International Consultation on Incontinence Questionnaire Anal Incontinence Symptoms and Quality of Life Module (ICIQ-B), a measure of anal incontinence symptoms and their impact on quality of life (25, 26); (3) the EuroQol 5-Dimensional (EQ-5D) questionnaire, a measure of health status (27); and (4) the Measure Yourself Medical Outcome Profile (MYMOP2), a measure of symptoms of high importance to the patient and their impact on activities of daily living (28). One participant, who runs a well-established late sequelae clinic for patients with pelvic organ cancers, also advocated for collecting measures of bladder and sexual dysfunction, and pain. The LARS score was reported to be the most commonly used PROM, where some participants used it to assess symptom severity, and others to identify patients with major LARS.

The format of data collection varied based on patient and clinician preferences, but in general, both paper and electronic formats were described. Some attested to providing patients with a paper or electronic form to fill out, which in most cases, comprised a combination of questions related to bowel function and PROMs. Data collection was less formalized for others, who would recommend that patients keep track of their symptoms with narrative comments or notes in free text.

#### Considerations for timing and duration

There was no agreed upon timing or duration of data collection for LARS patients, but most participants felt that the first several months following ileostomy closure were the most helpful, as this was when patients' symptoms were the most bothersome and distressing. During this time, clinicians felt that patients were establishing a new baseline and learning how to manage their symptoms. Based on their experience, KI14, a LARS Nurse, felt that in about 70% of patients, symptoms tended to stabilize after four to five months, after which patients had an improved understanding of their symptoms and associated triggers, making additional data collection less helpful.

In terms of bowel diaries, some of the informants felt that a minimum of two weeks of continuous data collection was necessary to be able to decipher what was going on with the patient. KI11, a LARS Nurse, emphasized the importance of having patients collect their data for multiple days in a row. This nurse preferred to ask patients to collect data two weeks prior to a visit, as opposed to continuously for months at a time between visits. KI01, a Colorectal Surgeon from a different institution, agreed:

I think two weeks is a good time. I think it's draining to do more than that. I think one to two weeks when you make a change. I always tell patients to do data collection for one to two weeks prior to the visit so they have something to talk about.—KI01, Colorectal Surgeon

In general, participants reported a duration of two weeks to be an appropriate amount of time to collect PGHD after implementing a new management strategy, such as a dietary change or fiber supplementation, to determine whether the intervention was effective.

### Data review practices

#### Variable approaches for reviewing data

Participants highlighted ways in which data were reviewed in the clinical environment, with varied approaches based on setting and clinician availability. Both surgeons and nurses attested to reviewing PGHD in the clinical setting. For the most part, participants endorsed reviewing data during clinical encounters, alongside patients. However, in some instances, nurses recalled reviewing data before or after the clinical encounter, depending on data availability. In one setting, the data being collected from patients was submitted electronically and reviewed remotely and asynchronously by the clinician:

So it's an electronic format; primarily electronic. And I think this is why we reach such a high number [of patients]. It's also electronically received by us. So we can run through the answers and find the responses which flag out. And on the basis of this response, then the nurse will make contact with the patient.—KI04, Colorectal Surgeon

Some participants described nurses as the primary providers reviewing PGHD with patients. Other informants stated that they do not review the data themselves, instead, it serves as an adjunct for patients to provide a history. For those who reviewed PGHD routinely in clinic, the time spent reviewing data was variable, but most quoted approximately 10–15 min.

Documentation and record keeping of PGHD also tended to vary by participant. Some informants attested to scanning a copy of the data and uploading it into the patient's electronic medical record (EMR), while others preferred to summarize pertinent findings in their clinic note.

#### Challenges associated with reviewing data

Informants highlighted several challenges associated with reviewing PGHD in the clinical setting, most notably, time constraints, legibility, completeness, interpretability, and compliance. For most participants, time was cited as the main barrier to reviewing patient data in clinic, where participants felt that they did not have sufficient time to spend reviewing data, especially during surveillance visits. Many expressed feeling rushed and referred to their clinics as being very busy, which in most cases, was not conducive to spending a significant amount of time reviewing data with the patient. When asked if reviewing PGHD hinders the clinical experience, KI01, a Colorectal Surgeon, replied:

No, but patients probably expected more out of me. They're polite. Nobody says anything, but if I did all this work, I probably wanted a bit more out of the clinician who's just like flipping through and saying, “Oh okay.” It's hard. It’s hard for me to digest it all and make recommendations sometimes based on it.—KI01, Colorectal Surgeon

Participants also pointed to legibility as an important barrier, and described situations where they could not decipher what had been recorded:

Some of the writing is so hard to decipher. And then they'll say, “Oh, I’ll do it,” and then they'll start reading word for word, and you're thinking, “Oh no, my consultation!” And you've got to squash it down.—KI12, LARS Nurse

This informant underscored a perceived loss of control when patients presented illegible data, potentially consuming more time than the clinician had to allocate to the appointment. Additionally, participants commented on the difficulty they experienced interpreting raw PGHD in the clinical setting. For example, one participant stated:

You know, they will have sat and recorded their stool movements for like 5 weeks. But then it's almost meaningless. It's just rows and rows and rows of type 3, type 4, three times a day. And it doesn't mean anything.—KI02, Colorectal Surgeon

This informant thought that it may be more efficient and effective to review bowel diary summaries, as opposed to reviewing the individual entries themselves. This was also discussed in the context of reviewing PROMs. In many cases, PROMs are reported as a summative number, and informants felt that it was sometimes difficult to put these numbers into context. For example, KI02, a Colorectal Surgeon stated: “It's very difficult sometimes for staff to look at and know what a score of 18 or 35 means.” Finally, many informants stated that a lack of consistency in data collection habits was a significant challenge. They felt that incomplete or inconsistent data collection made it difficult to interpret PGHD.

### Perceived usefulness

#### Helps clinicians understand patient symptoms

Some participants felt that the use of PGHD was helpful to understand patients' symptom burden, and identify patterns and trends in symptoms overtime, in turn, guiding management. KI06, a Colorectal Surgeon, elaborated on this and stated that PGHD helped them categorize patients by their predominant symptom and tailor their management accordingly:

I think it's very important to have data collection because a lot of the time it is really difficult for patients to tell us what is going on with them. It's, “oh my bowels are acting up” and then you start dialing in, they are never really able to give you a clear answer and part of it is because, it's not like patients with diarrhea or constipation where they have one consistent problem with their bowel habits. LARS tends to fluctuate a whole lot. And that's why a lot of our patients are not able to describe their symptoms at great length. […] We know it's LARS but are they having more issues with incontinence, are they having more issues with the frequency or clustering of bowel movements, or are they having more issues with urgency. […] It becomes a little bit easier, at least for me, to put them into those buckets. […] So you can modify the treatment that you offer, the modality that you offer to those patients.—KI06, Colorectal Surgeon

This surgeon expressed that they found PGHD particularly useful for LARS patients due to the variability in symptoms that patients experience and differences in the effectiveness of management strategies based on predominant symptoms. Being able to quickly identify predominant symptoms through the use of PGHD was thought to help inform management in an efficient way.

#### Assists patients with self-management

What appeared to be more significant, however, was the potential benefit of PGHD for patients themselves when used as a clinical tool. In many cases, participants felt that the use of PGHD helped engage patients in their care through self-reflection and self-management. It was believed that when patients collected data and tracked their health information, they gained a better understanding of their symptoms and were more likely to adopt management strategies. One informant described data collection and availability as a form of empowerment for the patient: “There is a lot of empowerment with certain groups of patients to have that data available. It does encourage self-management and awareness so they can see things changing” (KI02, Colorectal Surgeon). In many cases, participants also believed that the use of PGHD served as an adjunct to history taking, helping patients relay information on their symptom characteristics and impact. KI14, a LARS Nurse, pointed out that patients commonly forget their information when they're in front of their clinician: “Often, you know, you sit in front of the physician, or any health professional and you forget all your information. So, it's just a good reminder for the patient, for sure” (KI14, LARS Nurse). Additionally, few participants emphasized the potential benefit of PGHD in demonstrating clinical progress:

I think that if they keep track of it, you can point back and say, remember how 6 months ago, you were spending X time in the bathroom and you were in Depends. And now, we are at the point where you can leave your house, your pain is better controlled. Like, yes you are still struggling but you have gotten better. And that helps reorient people.—KI03, Colorectal Surgeon

This was described by another Colorectal Surgeon as “an objective record of treatment success” (KI02, Colorectal Surgeon).

However, the use of PGHD was not deemed beneficial for all patients, and informants cautioned against its use in certain cases. KI11, a LARS Nurse, recounted instances where patients had obsessively tracked their health information, generating an excessive amount of data. This participant believed that in such cases, data collection may hinder patient progress:

If I have patients who are obsessively tracking, sometimes I'll say, “Let’s just stop all of this.” They're writing down everything, two thirds of a cup of yogurt and all this stuff. And I'm like, “You need to back off of that a little bit and let’s think about big picture. Like mark it when you have an accident, mark it when you can’t get out of the bathroom, or whatever.” Rather than these kind of really aggressive things if patients are doing well.—KI11 LARS Nurse

In cases such as these, this informant would advise these patients to stop tracking and instead focus on the bigger picture. Similarly, another informant noted that patients who are not “data-driven” may feel overwhelmed by it, and consequently, would likely not benefit from it.

### Future directions

#### Optimal use of PGHD

There were differing perspectives on what types of data would be ideal to ask patients to collect. Some informants felt that a combination of PROMs, such as the LARS score, combined with granular information on bowel function, would be the most helpful. KI01, a Colorectal Surgeon, felt that it was important to obtain a summary measure of symptom severity, through a measure like the LARS score, as well as information on symptom characteristics and impact on quality of life, which could inform management strategies:

I think a combination of both. I think the LARS score just tells you about the degree or the pervasiveness of the problem in their day or in their week. […] But I think the granular data is what tells you where you can intervene.—KI01 Colorectal Surgeon

These forms of data were thought to complement one another and allow for a more complete clinical picture.

Several informants felt that an app or website as a form of patient-facing technology, with a clinician interface, would be helpful to facilitate data collection and review. However, many also highlighted the importance of ensuring the availability of alternative forms of data collection for patients who may not be technologically savvy. One participant stated:

I think for most of us younger people, having a phone in our hands, it's probably a lot easier just to record things on an app than trying to carry a piece of paper and a pen around. For a certain generation, paper and pen is easier. So, I think, having both options.—KI05 Colorectal Surgeon

Informants felt as though graphical displays or data visualizations could help both clinicians and patients understand patterns and trends in symptoms over time, and identify associations between symptoms and potential triggers. It was stated that this could optimize the value derived from the use of PGHD, and enhance its efficiency and effectiveness in clinical settings. Informants felt that data visualizations should be easy to interpret and include comprehensive summaries with information presented using graphical displays to facilitate understanding:

If I can get a summary or a dashboard where I see all the data in one glance, rather than having to go through all of those sheets… sheets after sheets of paper, and then trying to collate all of that data and coming up with an analysis right on the spot when I’m in the middle of a busy clinic. I mean that would absolutely be helpful.—KI06 Colorectal Surgeon

Few participants felt that it would be ideal to integrate a clinical tool directly into the EMR, for example, through MyChart as part of EPIC.

Yeah, I mean, there's so much available on MyChart right now that the patients have as an app on their phone already. So, I mean ideally, it’d be nice to incorporate that directly. So, you have some platform that the patient fills out on their MyChart app, and it goes directly into their EPIC. I think that would be the most ideal because it kind of cuts out the middleman, so to speak.—KI07, Colorectal Surgeon

This integrated system was described as a convenient solution for patients and clinicians, alike, with the data and clinical information accessible in one place.

#### Opportunities for innovation

A few participants discussed downstream opportunities that may arise from collecting PGHD routinely, namely, the use of artificial intelligence (AI) to assist patients and guide treatment:

You could even have an AI system that would put all of this information together and trigger things of urgency to be reviewed, and maybe even the AI system down the road would be able to be trained on all of the management recommendations themselves. So, the patient would input the problem management stuff and the AI system would spit back the data based on things. I mean, there's almost already so much stuff going on about AI reading radiographs. So, reading an abdominal film is not as complicated as reading an MRI. So, like several steps down the road in technology, that would be really amazing.—KI07, Colorectal Surgeon

This was also raised as a potential future direction by KI04, a Colorectal Surgeon, who saw promise in leveraging PGHD to inform the creation of automated treatment recommendations for LARS self-management using AI. It was felt that this could be used for surveillance in LARS patients, where patients could learn to manage their symptoms somewhat independently using treatment algorithms that provide tailored feedback on management strategies based on patients' symptoms, with the ability to notify the clinician of the presence of red flag symptoms:

It's also quite good as a prompt for patients who are in surveillance programs who don't necessarily need to be seen all the time but you sort of flag a warning and say, right—this is your 3 month LARS score and it's looking like things have changed. Do you want to see a consultant or do you want to see a professional. That's how I would like to use it.—KI02, Colorectal Surgeon

Similarly, another participant noted: “They could have a treatment plan that actually doesn't require contact with the health system, which would be ideal, economically as well.”—KI16 LARS Researcher.

## Discussion

This study provides valuable insights into the use of PGHD in the management of LARS, identifying four key domains: data collection practices, data review practices, perceived usefulness, and future directions, each with associated themes. In terms of data collection, informants described varying approaches and types of PGHD, such as bowel diaries and PROMs, with most recommending data collection during the first several months following ileostomy closure when symptoms are most severe. The data review practices domain revealed variability in how data was reviewed and identified several challenges, including time constraints, legibility, completeness, interpretability, and patient compliance. Participants recognized the utility of PGHD in helping clinicians understand trends in patients' symptoms and supporting self-management. Lastly, informants highlighted the potential for future innovation, including the development of patient-facing apps and AI-driven treatment algorithms to optimize PGHD use.

The findings of this study align with broader trends seen in oncology. Within oncology, PGHD has largely been used to track disease-related symptoms, side effects and consequences of treatment, and their impact on quality of life and daily functioning (17). Research has shown that oncologists tend to underestimate patients' symptoms and their impact, which has prompted interventions involving symptom monitoring through the use of PROMs (29). For instance, a randomized controlled trial demonstrated the effectiveness of a symptom monitoring intervention in patients with advanced solid tumors undergoing outpatient chemotherapy. This intervention involved sending email alerts to the clinical team in cases of severe or worsening symptoms, leading to nursing interventions such as telephone counseling, medication adjustments, or referrals. Patients who received web-based symptom monitoring were found to have better health-related quality of life (HRQL), fewer emergency room (ER) visits, fewer hospitalizations, and longer durations of palliative chemotherapy (30). In their discussion, the authors pointed to enhanced clinician awareness and associated improvements in symptom management during routine oncology care as the most likely underlying mechanism conferring clinical benefits (30). Similar benefits have been observed in surgical oncology, particularly with at-home symptom monitoring post-operatively, resulting in significant symptom reductions for patients undergoing thoracic cancer surgery (31). The findings of our study echo these trends, indicating that clinicians perceive the use of PGHD in the management of LARS as beneficial for increasing awareness of patients' symptom burden and its impact on quality of life. One informant perceived PGHD as very beneficial due to the variability in LARS symptoms. In a 2017 systematic review, Keane et al. identified over 30 symptoms experienced by LARS patients (32). This variability makes symptom monitoring well-suited to LARS management. Moreover, the concept of remote symptom monitoring with alerts based on red flag symptoms was discussed by a few informants. While existing programs are currently limited, the benefits observed in oncologic settings suggest that remote symptom monitoring with built in notifications for worsening or red flag symptoms may prove to be very beneficial for LARS patients and should be investigated in the future.

Participants identified several potential barriers to reviewing PGHD in clinic, including time constraints, legibility, completeness, interpretability, and compliance. This is consistent with other barriers to the use of PGHD cited in the literature, including reliability and accuracy of data, forgetfulness of patients, and innate attitudes towards technology (33). Informants in our study described feeling rushed in clinic due to high-volume clinics and busy schedules, which suggests that the current clinical infrastructure may not support the current use of PGHD collection and review. Moreover, participants acknowledged the time commitment required from patients in collecting their data, and expressed disappointment for not being able to spend more time reviewing the data in clinic. This highlights a potential tension between the type of care that clinicians would like to provide and the demands of their clinical workload, prohibiting a comprehensive review of PGHD in the clinical setting. A similar experience was reported in a recent systematic review, which explored the impact of PGHD collection and review on the patient-clinician relationships in surgery and primary care, and found that PGHD improved communication between patients and their clinicians, however, patients expressed a desire for more involvement from their clinicians (18). This underscores the importance of developing tools to enhance the efficiency of data collection and review practices, as well as gaining stakeholder buy-in to establish the infrastructure to support the implementation of such tools in clinical settings.

There are several limitations to this study. We used purposive sampling to recruit providers and research associates with a dedicated interest and expertise in LARS. As such, we collected a very narrow set of experiences and perspectives, which may not be reflective of colorectal surgery settings more broadly. Furthermore, we gained insights from international participants across five countries. However, it is important to acknowledge that the countries represented are high-income countries. Practices and resources in low- and middle-income countries may differ significantly, potentially affecting the applicability of these findings to those settings. Finally, our sample only included health care providers and research associates, and not patients. Research in this field has demonstrated the importance of considering both patient and clinician preferences in the creation of tools that facilitate the collection and review of PGHD (10). Future research will focus on incorporating the patient perspective, as well as including a broader and more heterogeneous sample of informants from different practice settings.

## Conclusion

This study highlights gaps in the current use of PGHD in the management of LARS. Data collection practices, data review practices, perceived usefulness, and future directions were identified as domains, each with associated themes. There remains a significant amount of variability in the ways in which PGHD is currently used for LARS management. A clinical tool including various data collection templates and data visualization prototypes could help optimize its use in LARS patients. Future research will incorporate the patient perspective.

## Data Availability

The datasets presented in this article are not readily available because of participant confidentiality. Even with names removed from the transcripts, the details provided in the interviews could identify study participants. Requests to access the datasets should be directed to Marylise Boutros, BOUTROM3@ccf.org.

## References

[1] Bryant CLC Lunniss PJ Knowles CH Thaha MA Chan CLH. Anterior resection syndrome. Lancet Oncol. (2012) 13(9):e403–408. 10.1016/S1470-2045(12)70236-X 22935240

[2] Emmertsen KJ Laurberg S Madsen M R Nielsen H J Ovesen A U Salomon S Impact of bowel dysfunction on quality of life after sphincter-preserving resection for rectal cancer. Br J Surg. (2013) 100(10):1377–87. 10.1002/bjs.9223 23939851

[3] Chen TY-T Wiltink LM Nout RA Meershoek-Klein Kranenbarg E Laurberg S Marijnen CAM Bowel function 14 years after preoperative short-course radiotherapy and total mesorectal excision for rectal cancer: report of a multicenter randomized trial. Clin Colorectal Cancer. (2015) 14(2):106–14. 10.1016/j.clcc.2014.12.007 25677122

[4] Juul T Ahlberg M Biondo S Espin E Jimenez LM Matzel KE Low anterior resection syndrome and quality of life: an international multicenter study. Dis Colon Rectum. (2014) 57(5):585–91. 10.1097/DCR.0000000000000116 24819098

[5] Battersby NJ Juul T Christensen P Janjua AZ Branagan G Emmertsen KJ Predicting the risk of bowel-related quality-of-life impairment after restorative resection for rectal cancer: a multicenter cross-sectional study. Dis Colon Rectum. (2016) 59(4):270–80. 10.1097/DCR.0000000000000552 26953985

[6] Emmertsen KJ Laurberg S. Low anterior resection syndrome score: development and validation of a symptom-based scoring system for bowel dysfunction after low anterior resection for rectal cancer. Ann Surg. (2012) 255(5):922–8. 10.1097/SLA.0b013e31824f1c21 22504191

[7] Lorig KR Holman HR. Self-management education: history, definition, outcomes, and mechanisms. ann Behav med. (2003) 26(1):1–7. 10.1207/S15324796ABM2601_01 12867348

[8] Corbin J Strauss A. Managing chronic illness at home: three lines of work. Qual Sociol. (1985) 8(3):224–47. 10.1007/BF00989485

[9] HealthIT.gov. What are Patient-Generated Health Data?. Office of the National Coordinator for Health Information Technology (ONC), US Department of Health Human Services. Available online at: healthit.gov/topic/otherhot-topics/what-are-patient-generated-health-data (cited 2024 March 26)

[10] Rajabiyazdi F Perin C Oehlberg L Carpendale S. Exploring the Design of Patient-Generated Data Visualizations. Available online at: https://hal.archives-ouvertes.fr/hal-02861239 (cited 2024 March 22).

[11] Brzan PP Rotman E Pajnkihar M Klanjsek P. Mobile applications for control and self management of diabetes: a systematic review. J Med Syst. (2016) 40(9):210. 10.1007/s10916-016-0564-8 27520615

[12] Karway G Grando MA Grimm K Groat D Cook C Thompson B. Self-management behaviors of patients with type 1 diabetes: comparing two sources of patient-generated data. Appl Clin Inform. (2020) 11(1):070–8. 10.1055/s-0039-1701002 PMC6976334 31968384

[13] Choe EK Lee B Kay M Pratt W Kientz JA. Sleeptight: low-burden, self-monitoring technology for capturing and reflecting on sleep behaviors. Proceedings of the 2015 ACM International Joint Conference on Pervasive and Ubiquitous Computing—ubiComp ‘15. Osaka, Japan: ACM Press, 2015. p. 121–32. Available online at: http://dl.acm.org/citation.cfm?doid=2750858.2804266 (cited 2022 November 9).

[14] Moen A Brennan PF. Health@home: the work of health information management in the household (HIMH): implications for consumer health informatics (CHI) innovations. J Am Med Inform Assoc. (2005) 12(6):648–56. 10.1197/jamia.M1758 16049230 PMC1294036

[15] Ancker JS Witteman HO Hafeez B Provencher T Van de Graaf M Wei E. The invisible work of personal health information management among people with multiple chronic conditions: qualitative interview study among patients and providers. J Med Internet Res. (2015) 17(6):e137. 10.2196/jmir.4381 26043709 PMC4526906

[16] Purpura S Schwanda V Williams K Stubler W Sengers P. Fit4life: the design of a persuasive technology promoting healthy behavior and ideal weight. Proceedings of the SIGCHI Conference on Human Factors in Computing Systems. Vancouver, BC, Canada: ACM, 2011. p. 423–32. Available online at: https://dl.acm.org/doi/10.1145/1978942.1979003 (cited 2022 November 9).

[17] Jim HSL Hoogland AI Brownstein NC Barata A Dicker AP Knoop H Innovations in research and clinical care using patient-generated health data. CA A Cancer J Clinicians. (2020) 70(3):182–99. 10.3322/caac.21608 PMC7488179 32311776

[18] Lordon RJ Mikles SP Kneale L Evans HL Munson SA Backonja U How patient-generated health data and patient-reported outcomes affect patient–clinician relationships: a systematic review. Health Informatics J. (2020) 26(4):2689–706. 10.1177/1460458220928184 32567460 PMC8986320

[19] Gotz D Borland D. Data-driven healthcare: challenges and opportunities for interactive visualization. IEEE Comput Grap Appl. (2016) 36(3):90–6. 10.1109/MCG.2016.59 28113160

[20] Garfinkle R Loiselle CG Park J Fiore JF Bordeianou LG Liberman AS Development and evaluation of a patient-centred program for low anterior resection syndrome: protocol for a randomized controlled trial. BMJ Open. (2020) 10(5):e035587. 10.1136/bmjopen-2019-035587 32474427 PMC7264642

[21] Harji D Fernandez B Boissieras L Berger A Capdepont M Zerbib F A novel bowel rehabilitation programme after total mesorectal excision for rectal cancer: the BOREAL pilot study. Colorectal Dis. (2021) 23(10):2619–26. 10.1111/codi.15812 34264005

[22] Tong A Sainsbury P Craig J. Consolidated criteria for reporting qualitative research (COREQ): a 32-item checklist for interviews and focus groups. Int J Qual Health Care. (2007) 19(6):349–57. 10.1093/intqhc/mzm042 17872937

[23] Gale NK Heath G Cameron E Rashid S Redwood S. Using the framework method for the analysis of qualitative data in multi-disciplinary health research. BMC Med Res Methodol. (2013) 13(1):117. 10.1186/1471-2288-13-117 24047204 PMC3848812

[24] Thompson J. A Guide to Abductive Thematic Analysis. TQR. (2022). Available online at: Available online at: https://nsuworks.nova.edu/tqr/vol27/iss5/17/ (cited 2023 October 17).

[25] Cotterill N Norton C Avery KNL Abrams P Donovan JL. Psychometric evaluation of a new patient-completed questionnaire for evaluating anal incontinence symptoms and impact on quality of life: the ICIQ-B. Diseases of the Colon & Rectum. (2011) 54(10):1235–50. 10.1097/DCR.0b013e3182272128 21904138

[26] Cotterill N Norton C Avery KNL Abrams P Donovan JL. A patient-centered approach to developing a comprehensive symptom and quality of life assessment of anal incontinence. Dis Colon Rectum. (2008) 51(1):82–7. 10.1007/s10350-007-9069-3 18008106

[27] Rabin R Charro FD. EQ-SD: a measure of health status from the EuroQol group. Ann Med. (2001) 33(5):337–43. 10.3109/07853890109002087 11491192

[28] Paterson C. Measuring outcomes in primary care: a patient generated measure, MYMOP, compared with the SF-36 health survey. Br Med J. (1996) 312(7037):1016–20. 10.1136/bmj.312.7037.1016 8616351 PMC2350813

[29] Laugsand EA Sprangers MAG Bjordal K Skorpen F Kaasa S Klepstad P. Health care providers underestimate symptom intensities of cancer patients: a multicenter European study. Health Qual Life Outcomes. (2010) 8(1):104. 10.1186/1477-7525-8-104 20858248 PMC2949821

[30] Basch E Deal AM Kris MG Scher HI Hudis CA Sabbatini P Symptom monitoring with patient-reported outcomes during routine cancer treatment: a randomized controlled trial. J Clin Oncol. (2016) 34(6):557–65. 10.1200/JCO.2015.63.0830 26644527 PMC4872028

[31] Cleeland CS Wang XS Shi Q Mendoza TR Wright SL Berry MD Automated symptom alerts reduce postoperative symptom severity after cancer surgery: a randomized controlled clinical trial. J Clin Oncol. (2011) 29(8):994–1000. 10.1200/JCO.2010.29.8315 21282546 PMC3068055

[32] Keane C Wells C O’Grady G Bissett IP. Defining low anterior resection syndrome: a systematic review of the literature. Colorectal Dis. (2017) 19(8):713–22. 10.1111/codi.13767 28612460

[33] Zhu H Colgan J Reddy M Choe EK. Sharing patient-generated data in clinical practices: an interview study. AMIA Annu Symp Proc. (2016) 2016:1303–12. 28269928 PMC5333267

