# Supplementary material for: The use of patient-generated health data in the management of low anterior resection syndrome: a qualitative study
Source: Front Surg. 2024 Dec 19;11:1506688. doi: 10.3389/fsurg.2024.1506688 (PMC11693684; doi:10.3389/fsurg.2024.1506688)
Supplement: Supplementary file 1 [file Datasheet1.pdf]

**Table A1.** Semi-structured interview guide.

| **Topic** | **Question** |
|---|---|
| **Surgeons and Nurses** | |
| Experience treating LARS patients | Surgeons:<br>1. To start off, would you mind telling me about your practice?<br>• Probe: How long have you been working as a colorectal surgeon?<br>2. Do you treat patients with low anterior resection syndrome (LARS) following restorative proctectomy?<br>• Probe: How long have you treated patients with LARS?<br><br>Nurses:<br>1. To start off, can you tell me about your experience working with LARS patients?<br>• Probe: How long have you worked with LARS patients?<br>2. Specifically, in what capacity/context do you work with LARS patients? |
| Experiences with LARS patient-generated data | 3. Do any of your patients with LARS present to clinical encounters with patient-generated LARS data?<br>• Probes:<br>◦ What types of data? (Examples: Data on bowel trouble, medication use, diet; patient-reported outcome measures)<br>◦ In what format?<br>◦ Is this patient-initiated or do you ask them to collect their data?<br>4. What type of data related to LARS do you find most helpful/useful? Why?<br>5. What do you think is the optimal duration of data collection?<br>6. How do you currently use this data in your practice?<br>• Probes:<br>◦ Do you review the patient data before, during, or after the appointment? How long do you spend reviewing it?<br>◦ How do you interpret or make sense of the data? Looking for trends?<br>◦ Does this help you understand and manage their LARS symptoms? If so, how?<br>7. What are some challenges you face when reviewing patient-generated LARS data in clinic? |

| | |
|---|---|
| | <ul><li>Probe: What barriers prevent you from making better use of the data your patients collect (Examples: time, format of data, legibility, completeness, reliability, sharing from patient device)?</li></ul>8. Do you collect or keep a record of patient-generated LARS data? If yes, how? If no, why not?<br>9. How effective do you think patient-collected LARS data is in supporting a patient's ongoing care?<br>10. What do you think the optimal future tool for patient-generated LARS data is?<ul><li>Probes: What format (paper, app, etc.)? Are there any specific features you think would be useful?</li></ul> |
| **Researchers** | |
| Experience treating LARS patients | 1. To start off, can you tell me about your experience working with LARS patients?<ul><li>Probe: How long have you worked with LARS patients?</li></ul>2. Specifically, in what capacity/context do you work with LARS patients? |
| Experiences with patient-generated LARS data | 3. Based on your discussions with patients, what types of data do LARS patients collect?<ul><li>Probes:<ul><li>Data on bowel trouble, medication use, diet; patient-reported outcome measures?</li><li>In what format?</li><li>Is this patient- or clinician-initiated?</li></ul></li></ul>4. What type of data related to LARS do you think is most helpful/useful? Why?<br>5. What do you think is the optimal duration of data collection is?<br>6. Do you know how data is reviewed by the healthcare team at your institution?<ul><li>Probes:<ul><li>Does the healthcare team review the patient data before, during, or after the appointment? Who is usually involved in this?</li><li>Do you yourself review patient-generated LARS data? If so, in what context?</li><li>In your experience, do you think reviewing patient-generated data helps the healthcare</li></ul></li></ul> |

| | |
|---|---|
| | team understand and manage patients' LARS symptoms? If so, how?<br>7. Does the healthcare team collect or keep a record of patient-generated LARS data? If yes, how? If no, why not?<br>8. How effective do you think patient-collected LARS data is in supporting a patient's ongoing care?<br>9. What do you think the optimal future tool for patient-generated LARS data is?<br>• Probes:<br>• What format (paper, app, etc.)?<br>• Are there any specific features you think would be useful? |
